# Buprenorphine–cannabis interaction in patients undergoing opioid maintenance therapy

**DOI:** 10.1007/s00406-019-01091-0

**Published:** 2020-01-06

**Authors:** Christopher Vierke, Brigitte Marxen, Michael Boettcher, Christoph Hiemke, Ursula Havemann-Reinecke

**Affiliations:** 1 Department of Psychiatry and Psychotherapy, University Medical Center Göttingen (UMG), Von Siebold Straße 5, 37075 Göttingen, Germany; 2 MVZ Dessau Laboratory GmbH, Bauhüttenstraße 6, 06847 Dessau, Germany; 3 Department of Psychiatry and Psychotherapy, University Medical Center Mainz, Mainz, Germany; 4 DFG Research Center of Nanoscale Microscopy and Molecular Physiology of the Brain (CNMPB), Humboldtallee 23, 37073 Göttingen, Germany

**Keywords:** Buprenorphine plasma level, Metabolic ratio, Cannabis, THC, Drug interaction, Opioid maintenance therapy, Gender

## Abstract

Buprenorphine is a partial μ-opioid agonist widely used for opioid maintenance therapy (OMT). It is mainly metabolized to pharmacologically active norbuprenorphine by the cytochrome P450 (CYP) isozyme 3A4. This may give rise to drug–drug interactions under combinations with inhibitors or inducers of CYP3A4. Cannabis is a potential inhibitor of CYP3A4, and there is a large degree of concomitant cannabis use among OMT patients. We performed a retrospective analysis on liver healthy OMT patients substituted with buprenorphine, either with (*n* = 15) or without (*n* = 17) concomitant use of cannabis. Patients with additional illicit drugs or medications affecting CYP3A were excluded. Measured blood concentrations of buprenorphine and norbuprenorphine were compared between the two groups. Cannabis users and non-users received similar doses, but users had 2.7-fold higher concentrations of buprenorphine (*p* < 0.01) and 1.4-fold for norbuprenorphine (1.4-fold, *p* = 0.07). Moreover, the metabolite-to-parent drug ratio was 0.98 in non-users and 0.38 in users (*p* = 0.02). Female gender did not produce significant effects. These findings indicate that cannabis use decreases the formation of norbuprenorphine and elevates buprenorphine and norbuprenorphine concentrations in blood most probably by inhibition of CYP3A4. The pharmacokinetic interaction may give rise to enhanced or altered opioid activity and risk of intoxications. Physicians should inform patients about this risk and supervise cannabis users by regular control of buprenorphine blood levels, i.e., by therapeutic drug monitoring.

## Introduction

In the past years, the prevalence of cannabis consumption has steadily increased with differential Δ9-tetrahydrocannabinol (THC) content in different preparations [1]. Patients undergoing opioid maintenance therapy (OMT) represent a traditional high-risk group for additional substance use and substance use disorders, [2] including cannabis. There is evidence that cannabinoids may interfere with the oxidative metabolism via the cytochrome P450 (CYP) isozymes and precipitate drug–drug interactions [3, 4]. The partial μ-opioid agonist buprenorphine is widely used for opioid maintenance therapy (OMT). Its long elimination half-life is subject to great inter-individual variation [5–7]. Elimination includes *N*-dealkylation to norbuprenorphine by CYP3A4, and to a lesser extent by CYP2C8, glucuronidation and biliary excretion [8]. Approximately 10–30% are excreted via the urinary tract [5, 7, 9]. As norbuprenorphine has a much longer elimination half-life than buprenorphine, patient adherence to the therapy regimen is associated with metabolite-to-parent drug concentration ratios greater than 1 in the presence of normal hepatic metabolism [10]. As norbuprenorphine is a much less potent analgesic drug than buprenorphine, sufficiently high buprenorphine plasma concentrations are required for the suppression of withdrawal symptoms and successful OMT [9]. Moreover, relapse prevention depends on sufficiently high drug concentrations [11–13]. To optimize efficacy and tolerability and to control buprenorphine’s susceptibility to critical drug–drug or food–drug interactions [9, 10, 14], therapeutic drug monitoring (TDM) should be applied [8]. Analysis of buprenorphine and norbuprenorphine in blood may support the clinician regarding the need for changes in drug dosage or administration patterns and enables detection of non-compliance. In particular, the drug concentration-to-dose ratio in conjunction with the metabolite-to-parent drug ratio presents a valid parameter for assessing compliance [15, 16].

To prevent intravenous abuse, sublingual formulations containing buprenorphine/naloxone combinations are available. However, there is still a large degree of illicit concomitant drug use in patients undergoing OMT. In a representative sample of 2694 German patients undergoing OMT, Wittchen and colleagues found concomitant use of at least one drug in 54% of patients [2]. Moreover, there is a high prevalence of hepatitis C virus (HCV) and human immunodeficiency virus (HIV) infection; in the Wittchen et al. cohort, 67% of patients tested positive for HCV and for 7.3% for HIV infection [2]. Hepatic inflammation, damage and impairment induced by HCV infection may interfere with the metabolism of various drugs, including buprenorphine; elevated levels of buprenorphine with decreased metabolism to norbuprenorphine in patients with hepatic impairment due to HCV infection have been reported [17]. Furthermore, the various antiviral medications used to treat HCV or HIV infection have been described to both inhibit and induce CYP enzymes, most notably CYP3A4. As a consequence, suboptimal levels of concurrent psychotropic medication have been found with a higher incidence in HIV/HCV-positive cohorts than in negative cohorts [18]. Wittchen and colleagues identified concomitant cannabis use in 30–46% in small and large OMT centers, respectively [2]. A study of the European Monitoring Centre for Drugs and Drug Addiction (EMCDDA) in 2015 revealed a one year prevalence of cannabis use of 5% in Europe and 10% in North America [19]. Significant psychiatric and somatic co-morbidity in patients suffering from addiction are a further complication in the management of these patients. Elevated buprenorphine levels were only found in patients with HCV infection plus moderate-to-severe liver impairment [20, 21]. Another risk factor is antiviral therapy with drugs known to cause interactions via the CYP isozymes [18, 22]. The CYP3A4-dependent metabolism of buprenorphine may be subject to interactions with both therapeutic and illicit drugs. This might put OMT patients at risk of altering their buprenorphine levels in a detrimental manner. Controlling the drug concentrations in blood by TDM can prevent buprenorphine-induced toxicity or relapse due to supra- or sub-therapeutic drug concentrations.

To evaluate a possible interaction between cannabis use and buprenorphine OMT, we analyzed serum buprenorphine and norbuprenorphine levels in patients without HCV and HIV infections undergoing OMT and supervised by TDM by comparing drug and metabolite concentrations in blood of cannabis users and non-users.

## Methods

### Patients

Patients undergoing OMT with either buprenorphine or buprenorphine/naloxone at the clinic for psychiatry and psychotherapy from 2012 to 2018 of the University Medical Center Göttingen (UMG) were included based on the following criteria: Patients had to be in buprenorphine OMT for at least 5 years and considered to be clinically stable by the treating physician. Stable buprenorphine dosage and take-home prescription constituted further inclusion criteria. Correct buprenorphine dosage was determined by clinical parameters (opioid withdrawal score, patient reporting well-being without feeling of “high”) with daily intake under supervision over a long time period before being approved for take-home prescription. Patients had to visit the clinic for supervised intake in the morning once a week. All patients with take-home prescription were instructed to take in the drug in the morning only. Patients acknowledged this as they required adequate buprenorphine dosing for their work. No consumption of any legal or illegal drugs except for cannabis for at least one year, no HIV infection, no active hepatitis with hepatic impairment and no medications with known CYP3A4 and CYP2C8 interactions constituted further inclusion criteria. All patients included in this work gave informed consent to TDM and scientific use of generated data.

### Laboratory measurements

For recording of trough plasma levels of buprenorphine, the blood samples were withdrawn 30 min prior to ingesting the next dose under supervision. Viral status for HCV, HBV and HIV was evaluated serologically by immunoassay, while medications were assessed by anamnesis and gas chromatography with mass spectroscopy/MS (GC/MS) in urine. We excluded patients with renal impairment as measured by plasma creatinine elevation.

### Liver function

For estimation of hepatic impairment or irritation, we used plasma transaminase activities (AST, ALT), γ-GT, INR and plasma albumin concentration to prevent falsely high buprenorphine concentrations due to HCV-induced liver damage or irritation. Transaminase and bilirubin elevations, INR prolongation and abnormal γ-GT constituted exclusion criteria.

### Drug, alcohol and medication use

Patients with a history of alcohol misuse were routinely tested for relapse by monitoring urine ethyl glucuronate, ethyl sulfate and serum carbohydrate-deficient transferrin by immunoassay. All patients were screened for drug misuse, including ethanol using immunologic urine testing on a monthly basis (Diagnostik Nord GmbH, see Table 1 for cutoff values) with subsequent confirmation of positive results for amphetamine and/or detection of other psychoactive drugs, using gas chromatography–mass spectrometry (GC/MS) or liquid chromatography–mass spectrometry (LC/MS). Furthermore, toxicological serum analysis using GC/MS was used for drug and medication testing both initially and punctually if warranted by clinical indication.

**Table 1:** Cutoff values for immunological drug screening in urine

Drug	Cutoff
Amphetamine	500 ng/mL
Cannabis (THC and metabolites)	50 ng/mL
Opiates	300 ng/mL
Cocaine	300 ng/mL
Benzodiazepines	300 ng/mL
Barbiturates	300 ng/mL
Methadone	300 ng/mL

### Measurement of cannabis/cannabinoids

The immunoassay (urine stick by Diagnostik Nord) qualitatively detects cannabis in urine on the base of THC and its metabolites. To assess possible dose-dependent effects of cannabis, an immunoassay was used to grossly determine urinary cannabinoid levels on the base of THC and the THC metabolites in a semiquantitative fashion, a CEDIA system (Microgenics, Freemont) in patients selected by clinical need. The test was sensitive for tetrahydrocannabinol (THC) and most of its known metabolites, intermediary for cannabinol but not for cannabidiol. This test did not differentiate between the different cannabinoids. The GC/MS method in urine qualitatively detects THC and metabolites, cannabinol and cannabidiol (CBD) or synthetic cannabinoids. Patients self-reporting once-daily cannabis smoking underwent punctual measurements of THC, 11-OH THC and 9-nor-carboxy-THC concentrations in serum using GC/MS to quantitatively estimate actual cannabinoid exposure in our patients.

### Serum buprenorphine measurements

For quantification of buprenorphine and norbuprenorphine in serum, a DIN EN ISO/IEC 17,025 accredited ultra-performance liquid chromatography–mass spectrometry method (Waters Acquity UPLC connected to TQ-S detector, Waters GmbH, Eschborn, Germany) was applied. Sample preparation: 100 µL serum was fortified with 20 µL internal standard solution containing 25 ng/mL buprenorphine-D4 (LGC Standards, Wesel, Germany) and 25 ng/mL norbuprenorphine-D3 (LGC Standards) in methanol. The sample was then protein-precipitated with 450 µL acetonitrile and 50 µL ammonia solution (32% v/v) and subsequently salted out with 50 µL 10 M ammonium acetate. After centrifugation, the organic supernatant was evaporated to dryness at 45 °C and the residue dissolved in 25 µL methanol + 125 µL mobile phase A. Injection volume into the UPLC-MS/MS system was 10 µL. Separation was conducted within 9 min on waters 2.1 × 150 mm, 1.8 µm HSS T3 column kept at 50 °C at a flow rate of 0.35 mL/min. Mobile phase A consisted of 20 mM ammonium formate (pH 3), and mobile phase B was 0.1% formic acid in methanol. Gradient separation started at 95% A and ended at 10% A. Data were acquired with an ESI source operating in the positive ionization, SRM mode with three transitions monitored per analyte and two transitions monitored per internal standard. Capillary voltage was set to 3 kV, ion source temperature was 150 °C, and desolvation gas was heated to 650 °C and delivered at a flow rate of 650 L/h. Cone gas flow (N_2_) was 150 L/h, and the collision gas flow (Ar) was 0.22 mL/min. The following transitions were monitored: buprenorphine: 468.2 > 396.2 (target ion), 468.2 > 414.3 (qualifier 1 ion), 468.2 > 83.8 (qualifier 2 ion); buprenorphine-D4: 472.3 > 400.2, 472.3 > 414.9, 4; norbuprenorphine: 414.3 > 83.1, 414.3 > 101.2, 414.3 > 187.1; norbuprenorphine-D3: 417.2 > 83.1, 417.2 > 152.2. Matrix calibration in human serum was performed at 0.1, 0.2, 0.3, 0.4, 0.5, 0.75, 1.0, 2.5 and 10 ng/mL. Limits of quantification were determined according to GTFCh guidelines at 0.1 ng/mL for buprenorphine and 0.2 ng/mL for norbuprenorphine. A commercial human serum control (STM 1–13-A SE, ACQ Science, Rottenburg-Hailfingen, Germany) and human urine control sample (FDT -25% UR, ACQ Science) with target values for buprenorphine at 3.51 ng/mL and 0.78 ng/mL and norbuprenorphine at 12.1 ng/mL and 0.74 ng/mL revealed CVs of 6.8% to 15.9% (*n* = 27).

### Statistics

Statistical analysis was conducted by Student’s *t* test to compare the cannabis and treatment group and assess possible gender effects with a significance level of *α* = 0.00625 as determined by Holm–Bonferroni method due to multiple testing. Eligible measurements were stratified for each patient. Mean dosage was calculated over the course of the years between 2012 and 2018. Mann–Whitney *U* tests, which does not require normal distribution, were performed. Multi-variant one-way ANOVA was employed due to evaluate possible effects of treatment duration. All statistical calculations were done using the Statistica Software version 13.3 for Windows by TIBCO Software Inc. (Palo Alto). Statistical values are reported as mean ± standard deviation unless specified otherwise. Graphical representations were created using Office Excel 2016 (Microsoft Corporation, Redmond).

### Drug interaction scoring

The probability of a cannabis–buprenorphine interaction was assessed using the Drug Interaction Probability Scale [23]. This scale employs 10 items to assess the probability of a drug interaction, with higher scores indicating higher probability of a drug–drug interaction. Scores between 2 and 4 are considered to indicate a possible interaction, 5–8 a probable interaction, while scores higher than 8 indicate a highly probable drug–drug interaction.

## Results

### Patient characteristics

After examining our patients for inclusion and exclusion criteria described above, we identified 32 eligible measurements out of a total of 79 serum buprenorphine measurements. Five patients with 17 measurements over a course of 5 years tested negative on toxicological screening for all drugs of abuse except nicotine, while 5 patients with 15 measurements, also over a course of 5 years, tested positive for cannabis only and were thus assigned to the cannabis group. A positive test for any cannabinoid measured by immunological test was considered a positive test for cannabis use. No patient reported cannabinoid prescription. In GC/MS analysis of urine, no intake of synthetic cannabinoid could be found in all patients. In all cases of cannabinoid detection by GC/MS, THC and metabolites were detected, seldom cannabinol, while CBD was never detected. All patients included were smokers. Three of the patients in the control group received buprenorphine/naloxone, while all patients in the cannabis group received buprenorphine-only preparations. Totally, 40% of our samples in the cannabis group came from female patients, while all samples from the control group came from male patients (Table 2). No difference in buprenorphine dosage was observed (Table 2). Mean age calculated as the arithmetic means of patient’s ages at the time the blood was drawn was 36.9 ± 9.9 years in the control group and 33.7 ± 3.3 years in the cannabis group. The last time of buprenorphine intake was documented for 53% of our patients and was 24.2 ± 2.1 h in the control group and 21.6 ± 5.1 h in the cannabis group. This difference was not significant (*p* = 0.18).

**Table 2:** Serum concentrations and dose-related values of cannabis non-users (control) and users

	Control (*n* = 17)	Cannabis (*n* = 15)	*p* (Student’s *t* test)	*p* (Mann–Whitney *U* test)
Dose	8.8 ± 3.9 mg	8.6 ± 0.9 mg	0.831	0.748
Serum buprenorphine	2.00 ± 3.17 ng/mL	5.41 ± 2.27 ng/mL	0.00167*	0.000249*
Serum norbuprenorphine	1.07 ± 0.82 ng/mL	1.76 ± 1.23 ng/mL	0.0694	0.08576
Active moiety	3.07 ± 3.53 ng/mL	7.17 ± 2.73 ng/mL	0.00103*	0.000632*
Metabolite-to-parent drug ratio	0.98 ± 0.78	0.39 ± 0.44	0.016	0.009171*
Concentration/dose ratio Buprenorphine	0.29 ± 0.36 ng/mL/mg	0.63 ± 0.26 ng/mL/mg	0.00519*	0.00223*
Concentration/dose ratio Norbuprenorphine	0.117 ± 0.077 ng/mL/mg	0.206 ± 0.147 ng/mL/mg	0.0372	0.0518
Active moiety/dose ratio	0.33 ± 0.34 ng/mL/mg	0.83 ± 0.32 ng/mL/mg	0.000141**	0.000268*

### Serum cannabinoid concentrations

Patients reported once-daily cannabis use at night had their serum cannabinoid levels measured, as outlined above. The concentrations of THC and its metabolites measured punctually were in the micromolar range: THC 14.29 ± 2.39 mg/L; THC-COOH 101.24 ± 56.32 mg/L; 11-OH-THC 1.97 ± 0.38 mg/L, *N* = 3. Considering the pharmakokinetics of smoked cannabis [24], these values indicate that we did not phlebotomize patients at the time of peak cannabinoid serum concentrations shortly after smoking. Overall, our observed concentrations are compatible with once-daily cannabis use by those patients. The urinary cannabinoid concentrations support frequent use of moderate amounts of cannabis.

### Dose and serum concentrations of buprenorphine and norbuprenorphine

The mean dose of buprenorphine in the control group was 8.8 ± 3.9 mg per day, while patients in the cannabis group received an average of 8.6 ± 0.9 mg a day. This difference was not significant with both *t* and Mann–Whitney *U* test. There were wide ranges of buprenorphine dosage in both groups due to different levels of opioid tolerance among patients (range 2.4–16 mg in the control group; 2–10 mg in the cannabis group). Within the control group, buprenorphine/naloxone (Suboxone®) therapy was not associated with significantly altered serum buprenorphine (*p* = 0.522) or dose-related concentrations compared to buprenorphine alone (Subutex®) (*p* = 0.504). Despite overall similar buprenorphine dosage, we found a highly significant correlation between cannabis use and serum buprenorphine level (*p* = 0.000563). Patients in the cannabis group reached a mean concentration of 5.4 ± 2.3 ng/mL (range 2.0–10.0 ng/mL; interquartile range 3.7–7.2 ng/mL), with control group patients reaching a mean of 2.0 ± 3.2 ng/ml (range 0.2–12.6 ng/mL; interquartile range 0.4–1.4 ng/mL).

Consistent with this, we found a significant correlation between cannabis use and the concentration/dose (C/D) ratio for buprenorphine. The C/D ratio for buprenorphine in the cannabis group was elevated (0.63 ± 0.54 vs 0.29 ± 0.36; *p* = 0.0019). The Mann–Whitney *U* test, which does not require normal distribution of data, confirmed the statistically significance of the Student’s test. On average, norbuprenorphine concentrations were higher in the cannabis group; however, this effect was not statistically significant. The metabolite-to-parent drug ratio was reduced in the cannabis group (0.98 ± 0.78 vs 0.39 ± 0.44; *p* = 0.016; see Table 3 for distribution). However, it was not significant.

**Table 3:** Distribution of metabolite-to-parent drug ratio in the control and cannabis group

	Control	Cannabis
Mean	0.423	0.18
SD	0.41	0.2
Median	0.93	0.278
Interquartile range Q1–Q3	0.33–1.33	0.13–0.42

Total active moiety was elevated in the cannabis group with a mean of 7.2 ± 2.7 ng/mL and a mean of 3.07 ± 3.54 ng/mL in the control group (*p* = 0.00103). The dose-related active moiety differed significantly as well, reaching 0.83 ± 0.32 ng/mL/mg in the cannabis group and 0.33 ± 0.34 ng/mL/mg in the control group.

In order to assess possible dose-dependent effects, we searched for a correlation of gross urinary cannabinoid concentrations as measured by immunoassay and serum buprenorphine levels and C/D ratios. We found a weak, but not significant positive correlation. However, in three of our patients not included in then original study due to HCV infection and co-medication who managed to permanently cease cannabis use with laboratory confirmation, serum buprenorphine, metabolic ratio and C/D ratios declined after cannabis cessation, supporting the idea of an interaction between cannabis use and buprenorphine metabolism.

### Gender effects

As 40% of measurements in the cannabis group came from female patients while the control group consisted of male patients only, we compared men and women in the cannabis group by performing multiple t-tests to assess effects of gender on the sample. We did not find a significant effect (Table 4). However, mean serum buprenorphine levels, metabolite-to-parent ratio and dose-related buprenorphine concentrations were slightly higher in female patients which is consistent with the findings reported by other investigators [25].

**Table 4:** Serum concentrations of buprenorphine, norbuprenorphine in male and female consumers of cannabis

	Male (*n* = 9)	Female (*n* = 6)	*p* (Student’s *t* test)	*p* (Mann–Whitney *U* test)
Dose	8.56 ± 1.0 mg	8.6 ± 0.81 mg	0.827	0.850
Serum buprenorphine	4.49 ± 1.97 ng/mL	6.80 ± 2.11 ng/mL	0.0495	0.0518
Serum norbuprenorphine	1.49 ± 1.54 ng/mL	2.17 ± 0.35 ng/mL	0.313	0.138
Metabolite-to-parent drug ratio	0.429 ± 0.58	0.34 ± 0.94	0.717	0.316
Active moiety	5.98 ± 2.4 ng/mL	8.97 ± 2.29 ng/mL	0.0316	0.0518
Concentration/dose ratio Buprenorphine	0.52 ± 0.21 ng/mL/mg	0.79 ± 0.26 ng/mL/mg	0.0421	0.0518
Concentration/dose ratio norbuprenorphine	0.17 ± 0.18 ng/mL/mg	0.25 ± 0.059	0.324	0.195
Dose-related active moiety	0.69 ± 0.26 ng/mL/mg	1.05 ± 0.30 ng/mL/mg	0.0308	0.0677

### Treatment effects

**Fig. 1:**
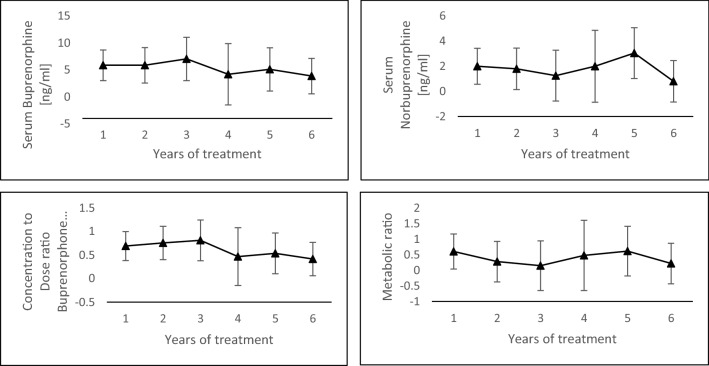
Mean serum buprenorphine of the cannabis group (*N* = 15), dose-related buprenorphine concentrations, serum norbuprenorphine concentrations and metabolite-to-parent drug ratios per years of measuring (error bars denote 95% confidence intervals)

In order to rule out effects of the duration of OMT and cannabis use, we performed multiple variant one-way ANOVA. There was no significant interaction in neither control group (Wilken’s *Λ* = 0.266, *p* = 0.8923) nor the cannabis group (Wilken’s *Λ* = 0.006, *p* = 0.152) nor in a group of pooled patients from both groups (Wilken’s *Λ* = 0.292, *p* = 0.9312; see Fig. 1).

### Case report

One patient in long-term OMT who was followed in this study since 2012 being clinically stable and in long-term employment ever since presented with marked clinical decline. He had always been using cannabis while on OMT with no additional concomitant drug use and required gradual increases in buprenorphine dosage from 7 to 10 mg daily over a course of 6 years starting 2012. This was consistent with increasing physical requirements by his occupation. He did not require any medication except for a pregabalin prescription (300 mg/d) which is not known to interfere with buprenorphine or cannabis metabolism. At one presentation, he reported sleep disturbances, agitation and decreased motivation, drive, contemplation and overall mood. Affect lability was found to be increased, and a depressive episode was diagnosed. Furthermore, the patient reported he had quit cannabis use a month ago, and this was confirmed by immunologic urine analysis and serum GC/MS for THC and metabolites (serum THC < 0.5 ng/mL, THC-COOH < 2.5 ng/mL, 11-OH-THC < 0.5 ng/mL) compared to presentation 6 months earlier (THC 16.82 ng/mL, THC-COOH 28.46 ng/mL, 11-OH-THC 1.63 ng/mL). He reported improved feeling of somatic health but increasing psychological issues. Upon clinical observation, he appeared pale, sweaty and agitated, indicating a withdrawal syndrome. The patient reported severe buprenorphine craving; this prompted buprenorphine status evaluation. Indeed, both buprenorphine and norbuprenorphine levels were markedly decreased compared to presentation six months earlier (0.3 ng/mL vs 6.3 ng/mL for buprenorphine; 0.3 ng/mL vs 1.0 ng/mL for norbuprenorphine), metabolic ratio having increased (1.00 compared to 0.16 6 months earlier). The patient was started on supportive therapy for psychic instability, depressed mood and insomnia using valproic acid (600 mg ramp-up) and mirtazapine (30 mg) and buprenorphine dosage was increased to 12 mg daily. Upon re-evaluation 14 days later, his clinical condition stabilized, while buprenorphine craving and agitation were still increased, albeit greatly ameliorated. The patient did not appear pale anymore. Serum buprenorphine concentration and dose-related concentrations were increased (2.1 ng/mL and 0.2 ng/mL/mg for buprenorphine; 0.8 ng/mL and 0.1 ng/mL/mg for norbuprenorphine; metabolic ratio 0.38); however, both plasma levels and dose-related concentrations had not rebounded to levels prior to cannabis cessation. This effect was consistent with prior attempts of this patient to reduce cannabis use with reductions in dose-related buprenorphine concentrations occurring at those times.

### Drug interaction score

In the evaluation using the Drug Interaction Probability Score (DIPS) proposed by Horn and co-workers, the interaction was rated as ‘probable’, as intra-individual comparison in three patients who managed to quit cannabis use altogether showed all a decline of buprenorphine levels after cannabis cessation. This is consistent with the findings of a fourth patient described above, giving rise to a drug interaction score of probable.

## Discussion

This retrospective analysis of buprenorphine concentrations in blood of opioid-dependent patients found elevated buprenorphine levels and metabolite-to-parent drug ratios in the cannabis patients compared to the control group. This suggests a lowered metabolic rate possibly via CYP3A4, CYP2C8 and/or the UGT2B7 pathways. Since CYP3A4 is the most important enzyme responsible for *N*-dealkylation of buprenorphine [5, 8], CYP3A4 inhibition may be assumed. Intra-individual comparison in patients who managed to quit cannabis use showed a decline of buprenorphine levels after cannabis cessation, giving rise to a drug interaction score of probable. This is consistent with the literature findings: Damkier et al. described the case of a 27-year-old male undergoing warfarin therapy due to endocarditis with subsequent mechanic heart valve replacement with severe INR elevation after recreational cannabis administration [26]. Additional similar case reports exist [27]. These reports are in line with findings in our own patients, one report described above. This underlines that changes in pharmacokinetics precipitated by cannabis are to be reckoned with by the treating physician, as changes in cannabis consumption habits may necessitate changes in treatment. Considering increasing prevalence of cannabis use, this has implications beyond the scope of addiction medicine, being relevant for the treatment of psychoses, depression and cancer, to name a few.

Cannabis is a heterogeneous substance with great variability in its active ingredients [1, 28]. In vitro data suggest an inhibitory effect of cannabidiol (CBD), but not THC on CYP3A4 (IC50 = 1 μM) (reviewed by [4]). Both CBD and cannabinol (CBN) as well as THC seem to inhibit CYP2C9 (IC50 = 0.95–9.88 µM for CBD, IC50 = 0.88–1.29 μM for CBN, IC50 = 0.94–1.50 μM for THC, respectively). Especially drug–drug interactions precipitated by CBD have gained interest as CBD is being employed in the treatment of refractory epilepsia [29, 30]. There is further evidence for CBDs efficacy as an antipsychotic [31–34] and its usefulness in the treatment of non-motor symptoms in Parkinson’s disease [35]. CYP3A4 inhibition by CBD may explain the increased buprenorphine levels observed. CYP2C8 inhibition may exacerbate this effect, as there are FDA caveats against CYP2C8 inhibition by CBD [36, 37]. As both CBD and buprenorphine are substrates of uridyl-glucuronosyl transferase 2B7 (UGT2B7), competition for this transporter may impair fecal buprenorphine excretion, further increasing plasma concentration. A virtually unlimited amount of cannabis strains is available on the black market, including strains with significant CBD content. In the past decades, overall content of psychoactive cannabinoids in the cannabis plant in both the USA and the European Union has increased, especially the content of THC [1]. Several medicinally available strains of cannabis contain high CBD levls. This poses a significant risk of patients reliant on any drug subject to CYP3A4-mediated metabolism, especially considering the increasing prevalence of both illicit and medical cannabis use, legalization and the fact that high CBD cannabis tends to be marketed as especially healthy. An interaction between clobazam and CBD has been reported [29, 38] by Geffrey, Pollack et al. in children being treated for refractory epilepsy; elevated clobazam and norclobazam levels were found, precipitating side effects requiring a dose reduction.

Although the in vitro data favor the role of CBD but less of THC on buprenorphine plasma levels, our clinical in vivo data, underlined by the case report with quantitative measurement of THC and its metabolites, lead to the suspicion that THC may probably also have inhibitory effects on CYP3A4. The buprenorphine levels decreased with decreasing THC levels. However, we did not assess serum CBD levels. Further studies have to be conducted to clarify the role of different substances of cannabis on the CYP isozymes in vivo.

To avoid interactions with other drugs, we eliminated confounding effects of co-medication by excluding patients under medications with known inhibitory or inducing potential on CYP3A4 or CYP2C. Most psychiatric patients, however, require several drugs which are substrates, inducers or inhibitors of the CYP isozymes including CYP3A4 and CYP2C9, both inhibited by CBD (quetiapine, fluoxetine, levomepromazine [10]), leading to unpredictable buprenorphine levels. Consumption of readily available CYP inducers or inhibitors like St. John’s Wort or excessive amounts of grapefruit juice, respectively, may exacerbate this issue. The same holds true for patients receiving cannabis for chronic pain, or for chemotherapy-induced nausea. The serum levels of THC, hydroxylated THC (11-OH-THC) and THC-carboxylic acid (THC-COOH) measured by us were comparable steady-state concentrations of frequent users of cannabis via smoking, inhalation and the oral route, including prescription drugs like Dronabinol® or Sativex® [24, 39]. Concordantly, these patients did not consume cannabis in extremely and uncommonly high amounts. Still, we only measured cannabinoid concentrations (THC and metabolites) in few patients; therefore, total cannabis exposure of other patients can only be estimated.

## Limitations

While we selected the cannabis users and non-users as control group with great care in regard to confounding factors, our approach harbors several limitations: Most of the patients were men, possibly inferring bias. There is evidence for higher CYP3A4 activity in women [25], warranting increased vigilance in regard to interactions. We did not find significantly higher buprenorphine levels or metabolite-to-parent drug ratios in female patients of our study, but recognized a trend for higher buprenorphine levels. This could be due to increased sensitivity to CYP3A4 inhibition due to increased CYP3A4 activities, but this remains unknown. Drug consumption was closely evaluated using GC/MS; however, patients were followed over several years: Since cannabis contents vary widely among different “suppliers” in regard to cannabinoid composition and total cannabinoid concentration, significant changes in cannabinoid consumption in the presence of normal test findings cannot be ruled out. Duration of buprenorphine treatment but also of concomitant cannabis use did not influence buprenorphine levels in our study. In vitro data suggest highly divergent action of different cannabinoids on the cytochrome P450 system [4]; therefore, different batches of cannabis may have divergent effects on serum buprenorphine concentrations. However, the immunoassay used in this study did not differentiate between the cannabinoids and was not sensitive for CBD as all other usually available cannabis tests. Furthermore, in the functionally performed urine tests with GC/MS of the cannabis user, we did not find CBD assuming that the preparations of cannabis used by the patients tested probably contained rather low concentrations of CBD. A standardized approach with defined cannabinoid intake is necessary to assess these effects in detail. We only included patients from a single center of care, increasing risk of bias in regard to the composition of the illegal cannabis used by our patients. Nasser et al. reported buprenorphine levels to be higher in subjects with HCV seropositivity or hepatic impairment [20, 21].

## Conclusion

Overall, increased serum buprenorphine levels and concentration-to-dose ratios support a cannabis–buprenorphine interaction. A decreased metabolite-to-parent drug ratio indicates reduced *N*-dealkylation, suggesting that cannabis preparations consumed by our patients inhibited CYP3A4 activity in favor of other metabolic pathways. Competition for UGT2B7 may lead to less buprenorphine conjugation and thus retention, further decreasing metabolite-to-parent drug ratio. This is consistent with preliminary in vitro and in vivo data. It should be taken into consideration when trying to wean OMT patients from cannabis use as it may cause additional buprenorphine withdrawal symptoms and may require increased buprenorphine dosage to stabilize these patients. Considering increasing use of cannabinoids in oncology and pain management, caution should be advised when prescribing medical cannabis preparations since a drug interaction could cause serious complications in settings of psychiatric treatment, pain treatment, chemotherapy or anticoagulation. The increasing recreational use of cannabis presents additional issues; physicians should be aware of this interaction, enabling them to give advise to their patients in order to maximize treatment efficacy and minimize side effects and drug toxicity precipitated by concomitant cannabis use by the patients unknown to the treating physician.

Therapeutic drug monitoring of opioids (here buprenorphine) seems to be a valuable methodological option for optimizing maintenance treatment in case of additional intake of interacting substances by reducing the risk of plasma-level-dependent toxicities and other undesirable effects.
